# Supplementary material for: Sleeping Disorders in Healthy Individuals with Different Dietary Patterns and BMI, Questionnaire Assessment
Source: Int J Environ Res Public Health. 2021 Nov 23;18(23):12285. doi: 10.3390/ijerph182312285 (PMC8656560; doi:10.3390/ijerph182312285)
Supplement: Supplementary file 1 [file ijerph-18-12285-s001.zip › File S1.pdf]

1. Jaką dietę Pan\Pani stosuje?

- Wegetariańska
- Wegańska
- Stosuję dietę konwencjonalną (z mięsem)

2. Od jak dawna stosuje Pan\Pani dietę wegetariańską lub wegańską?"

Jeżeli dietę wegańską poprzedzała wegetariańska, proszę podać czas od zrezygnowania ze spożywania mięsa. Osoby, które nie rezygnują z mięsa proszę o zaznaczenie odpowiedzi nie dotyczy".

- Poniżej 1 miesiąca
- 1-6 miesięcy
- 6-12 miesięcy
- 1-4 lata
- 5 lat lub więcej
- Nie dotyczy

3. Płeć *

- Kobieta
- Mężczyzna

4. Wiek *

5. Masa ciała w kilogramach *

6. Wysokość ciała w centymetrach *

Ocena czynników wpływających na jakość snu

7. Czy w dni robocze kładzie się Pan/Pani spać codziennie o podobnej godzinie? *

Możliwa rozpiętość godzin: około jedna godzina. Np. jeżeli codziennie kładzie się Pan/Pani między 21-22 proszę zaznaczyć odpowiedź tak.

- Tak
- Nie

8. Czy w dni robocze wstaje Pan/Pani codziennie o podobnej godzinie? *

Możliwa rozpiętość godzin: około jedna godzina. Np. jeżeli codziennie wstaje Pan/Pani między 7-8 proszę zaznaczyć odpowiedź tak.

- Tak
- Nie

9. Czy w dni wolne wstaje Pan/Pani o podobnej godzinie co w dni robocze? *

Możliwa rozpiętość godzin około jedna godzina.

- Tak

- Nie

10. Czy w dni wolne kładzie się Pan/Pani spać o podobnej godzinie co w dni robocze? *
Możliwa rozpiętość godzin około jedna godzina

- Tak
- Nie

11. Jak często robi Pan/Pani drzemki? *

- Nigdy
- 1-3 razy w miesiącu
- Raz w tygodniu
- Kilka razy w tygodniu
- Raz dziennie
- Kilka razy dziennie

12. Jeżeli robi Pan/Pani drzemki to ile minut zazwyczaj trwają?
Proszę wpisać cyfrą ile minut trwają drzemki.

13. Czy śpi Pan/Pani w zaciemnionej sypialni?
Na przykład czy ma Pan/Pani szczelne rolety zapobiegające dostępowi światła

- Tak
- Nie

14. Czy w godzinach dopołudniowych jest Pan/Pani wystawiony/a na silne światło (naturalne bądź sztuczne).

- Tak
- Nie
- Nie wiem

15. Czy w ciągu ostatniego roku zauważył/a Pan/Pani zmianę w jakości swojego snu w porównaniu do wcześniejszej jakości swojego snu?

- Tak
- Nie
- Trudno ocenić

16. Czy pali Pan\Pani papierosy?*

- Tak
- Nie

17. Czy pracuje Pan/Pani w trybie zmianowym ze zmianami nocnymi? *

- Tak

- Nie

18. Czy przyjmuje Pan/Pani obecnie leki nasenne lub leki o działaniu sedacyjnym? *

- Tak
- Nie

19. Czy przyjmuje Pan/Pani obecnie leki mogące wpływać na jakość lub ciągłość snu?"
Np. Rezerpina (Normatens), metyldopa (Dopegyt), klonidyna (Iporel), Propranolol (Propranolol Accord). Teofilina (Euphylin)

- Tak
- Nie

20. Czy przyjmuje Pan/Pani suplementy diety wspomagające zasypianie i sen? *

- Tak
- Nie

21. Jeśli przyjmuje Pan/Pani suplementy diety wspomagające zasypianie i sen, to jakie?

22. Czy cierpi Pan/Pani na zespół obturacyjnego bezdechu sennego? *

- Tak
- Nie

23. Czy cierpi Pan/Pani na zaburzenia psychiatryczne, które mogą wpływać na jakość snu?*
Np. Depresja, choroba afektywna dwubiegunowa, zespół lęku uogólnionego, zespół stresu pourazowego, schizofrenia

- Tak
- Nie

24. Czy cierpi Pan/Pani na jakieś schorzenie, któremu towarzyszy przewlekły ból?"

- Tak
- Nie

25. Czy korzysta Pan/Pani z urządzeń elektronicznych (telefon, tablet, komputer) na godzinę przed pójściem spać?

- Tak, praktycznie codziennie
- Tak, 3-4 razy w tygodniu
- Tak, 1-3 razy w tygodniu
- Tak, rzadziej niż 1 raz w tygodniu
- Nie

26. Czy ma Pan/Pani w urządzeniach elektronicznych włączony filtr/bloker światła niebieskiego lub nocny tryb czytania?

- Tak
- Nie
- Nie wiem

27. Czy używa Pan/Pani okularów z filtrem światła niebieskiego? *

- Tak
- Nie
- Nie wiem

28. Jak często pije Pan/Pani kawę 5 lub mniej godzin przed pójściem spać?*

- Nigdy
- 1-3 razy w miesiącu
- Raz w tygodniu
- Kilka razy w tygodniu
- Codziennie

29. Jak często pije Pan/Pani alkohol 3 lub mniej godzin przed zaśnięciem? *

- Nigdy
- 1-3 razy w miesiącu
- Raz w tygodniu
- Kilka razy w tygodniu
- Codziennie

30. Gdy pije Pan/Pani alkohol przed snem, to zazwyczaj jaką ilość?

31. Jak często uprawia Pan/Pani intensywny sport 2 godziny przed zaśnięciem? *
Nie uwzględniamy tutaj jogi, lekkiego stretchingu czy ćwiczeń oddechowych.

- Nigdy
- 1-3 razy w miesiącu
- Raz w tygodniu
- Kilka razy w tygodniu
- Codziennie

32. Czy uważa Pan/Pani, że obecnie występuje w Pańskim życiu jakiś czynnik mogący wpływać negatywnie na jakość snu? Jeśli tak to jaki?

Ocena jakości diety

33. Ile posiłków spożywa Pan/Pani zazwyczaj w ciągu dnia?*

- 1 posiłek
- 2 posiłki
- 3 posiłki
- 4 posiłki
- 5 posiłków lub więcej

Proszę sobie przypomnieć swój typowy pod względem odżywiania dzień w ostatnim tygodniu, a następnie odpowiedzieć na kolejne pytania.

34. Jaki to był dzień tygodnia?

- Poniedziałek
- Wtorek
- Środa
- Czwartek
- Piątek
- Sobota
- Niedziela

35. Ile posiłków zjadł(a) Pan/Pani w tym dniu? Proszę wpisać liczbę posiłków w tym dniu.

36. Ile razy zjadł(a) Pan/Pani w tym dniu warzywa lub owoce? *

Proszę wpisać liczbę razy w tym dniu. Należy uwzględnić w posiłkach i między posiłkami

37. Czy zjadł(a) Pan/Pani w tym dniu żywność typu fast food, np. frytki, hamburgery, pizzę, hot dogi, *

- Tak
- Nie

38. Ile razy zjadł(a) Pan/Pani w tym dniu żywność typu fast food, np. frytki, hamburgery, pizze, hot-dogi, zapiekanki?

Jeżeli nie jadł(a) Pan/Pani takie żywności tego dnia proszę wpisać 0.

Częstotliwość spożycia żywności

Teraz zadam pytania dotyczące rodzaju żywności, którą Pan/Pani spożywa. Chcemy się dowiedzieć, jak często zazwyczaj jada Pan/Pan te żywność? Odpowiadają na pytania prosze uwzględnić żywność jadaną w posiłkach i między posiłkami, w domu poza domem w ciągu ostatniego roku w tej części proszę wskazać jedną odpowiedź.

39. Jak często spożywa Pan/Pani pieczywo jasne, np. pszenne, żytnie, mieszane pszenno-żytnie, pieczywo tostowe, butki, rogale?

- Nigdy
- 1-3 razy w miesiącu

- Raz w tygodniu
- Kilka razy w tygodniu
- Raz dziennie
- Kilka razy dziennie

40. Jak często spożywa Pan/Pani pieczywo razowe?

- Nigdy
- 1-3 razy w miesiącu
- Raz w tygodniu
- Kilka razy w tygodniu
- Raz dziennie
- Kilka razy dziennie

41. Jak często spożywa Pan/Pani ryż biały, makaron zwykły lub drobne kasze, np. kaszę mannę. kuskus?

- Nigdy
- 1-3 razy w miesiącu
- Raz w tygodniu
- Kilka razy w tygodniu
- Raz dziennie
- Kilka razy dziennie

42. Jak często spożywa Pan/Pani kaszę gryczaną, płatki owsiane, makaron pełnoziarnisty lub inne kasze gruboziarniste?

- Nigdy
- 1-3 razy w miesiącu
- Raz w tygodniu
- Kilka razy w tygodniu
- Raz dziennie
- Kilka razy dziennie

43. Jak często spożywa Pan/Pani zywność typu fast food, np, frytki, hamburgery, pizzę, hot dogi. zapiekanki?

- Nigdy
- 1-3 razy w miesiącu
- Raz w tygodniu
- Kilka razy w tygodniu
- Raz dziennie
- Kilka razy dziennie

44. Jak często spożywa Pan/Pani potrawy smażone (np. mięsne lub mączne)? *

- Nigdy
- 1-3 razy w miesiącu

- Raz w tygodniu
- Kilka razy w tygodniu
- Raz dziennie
- Kilka razy dziennie

45. Jak często spożywa Pan/Pani masło jako dodatek do pieczywa lub potraw, do smażenia. pieczenia itp.?

- Nigdy
- 1-3 razy w miesiącu
- Raz w tygodniu
- Kilka razy w tygodniu
- Raz dziennie
- Kilka razy dziennie

46. Jak często spożywa Pan/Pani smalec jako dodatek do pieczywa lub potraw, do smażenia, pieczenia itp.?

- Nigdy
- 1-3 razy w miesiącu
- Raz w tygodniu
- Kilka razy w tygodniu
- Raz dziennie
- Kilka razy dziennie

47. Jak często spożywa Pan/Pani mleko (w tym mleko smakowe, kakao, kawę na mleku)? *

- Nigdy
- 1-3 razy w miesiącu
- Raz w tygodniu
- Kilka razy w tygodniu
- Raz dziennie
- Kilka razy dziennie

48. Jak często spożywa Pan/Pani fermentowane napoje mleczne, np. jogurty, kefiry (naturalne, smakowe)?

- Nigdy
- 1-3 razy w miesiącu
- Raz w tygodniu
- Kilka razy w tygodniu
- Raz dziennie
- Kilka razy dziennie

49. Jak często spożywa Pan/Pani sery twarogowe (w tym serki homogenizowane, desery twarogowe)?

- Nigdy
- 1-3 razy w miesiącu
- Raz w tygodniu
- Kilka razy w tygodniu
- Raz dziennie
- Kilka razy dziennie

50. Jak często spożywa Pan/Pani sery żółte (w tym serki topione, sery pleśniowe)? *

- Nigdy
- 1-3 razy w miesiącu
- Raz w tygodniu
- Kilka razy w tygodniu
- Raz dziennie
- Kilka razy dziennie

51. Jak często spożywa Pan/Pani wędliny, kiełbasy lub parówki?

- Nigdy
- 1-3 razy w miesiącu
- Raz w tygodniu
- Kilka razy w tygodniu
- Raz dziennie
- Kilka razy dziennie

52. Jak często spożywa Pan/Pani potrawy z tzw. mięsa czerwonego, np. wieprzowiny, wołowiny, cielęciny, baraniny, jagnięciny, dziczyzny?

- Nigdy
- 1-3 razy w miesiącu
- Raz w tygodniu
- Kilka razy w tygodniu
- Raz dziennie
- Kilka razy dziennie

53. Jak często spożywa Pan/Pani potrawy z tzw. mięsa białego, np. z kurczaka, indyka, królika?

- Nigdy
- 1-3 razy w miesiącu
- Raz w tygodniu
- Kilka razy w tygodniu
- Raz dziennie
- Kilka razy dziennie

54. Jak często spożywa Pan/Pani ryby? *

- Nigdy

- 1-3 razy w miesiącu
- Raz w tygodniu
- Kilka razy w tygodniu
- Raz dziennie
- Kilka razy dziennie

55. Jak często spożywa Pan/Pani potrawy z nasion roślin strączkowych, np. fasoli, grochu, soi, soczewicy?

- Nigdy
- 1-3 razy w miesiącu
- Raz w tygodniu
- Kilka razy w tygodniu
- Raz dziennie
- Kilka razy dziennie

56. Jak często spożywa Pan/Pani świeże owoce? *

- Nigdy
- 1-3 razy w miesiącu
- Raz w tygodniu
- Kilka razy w tygodniu
- Raz dziennie
- Kilka razy dziennie

57. Jak często spożywa Pan/Pani warzywa?

- Nigdy
- 1-3 razy w miesiącu
- Raz w tygodniu
- Kilka razy w tygodniu
- Raz dziennie
- Kilka razy dziennie

58. Jak często spożywa Pan/Pani słodycze, np. cukierki, ciastka, ciasta, batony czekoladowe, batony typu 'musli", inne wyroby cukiernicze?)

- Nigdy
- 1-3 razy w miesiącu
- Raz w tygodniu
- Kilka razy w tygodniu
- Raz dziennie
- Kilka razy dziennie

59. Jak często spożywa Pan/Pani konserwy mięsne? *

- Nigdy
- 1-3 razy w miesiącu

- Raz w tygodniu
- Kilka razy w tygodniu
- Raz dziennie
- Kilka razy dziennie

60. Jak często pije Pan/Pani słodzone napoje gazowane lub niegazowane typu Coca-Cola, Pepsi, Sprite, Fanta, oranżada, lemoniada?

- Nigdy
- 1-3 razy w miesiącu
- Raz w tygodniu
- Kilka razy w tygodniu
- Raz dziennie
- Kilka razy dziennie

61. Jak często pije Pan/Pani napoje energetyzujące, np. 2 KC, Black Horse, Red Bull, Burn, Shot lub inne?

- Nigdy
- 1-3 razy w miesiącu
- Raz w tygodniu
- Kilka razy w tygodniu
- Raz dziennie
- Kilka razy dziennie

62. Jak często pije Pan/Pani napoje alkoholowe? *

- Nigdy
- 1-3 razy w miesiącu
- Raz w tygodniu
- Kilka razy w tygodniu
- Raz dziennie
- Kilka razy dziennie

Ocena występowania zaburzeń odżywiania

Ateńska skala bezsenności

63. Jak szybko zasypia Pan/Pani po położeniu się do łóżka i zgaszeniu światła? *

- Szybko
- Z nieznacznym opóźnieniem
- Z opóźnieniem
- Z bardzo dużym opóźnieniem lub bezsenność trwa całą noc

64. Czy budzi się Pan Pani w nocy?*

- Nie

- Sporadycznie
- Często
- Bardzo często lub bezsenność trwa całą noc

65. Czy budzi się Pan Pani rano wcześniej niż planowano? *

- Nie
- Nieznacznie wcześniej
- Znacznie wcześniej
- Dużo wcześniej lub bezsenność trwa całą noc.

66. Jaki jest całkowity czas Pańskiego snu? *

- Wystarczający
- Nieznacznie niewystarczający
- Znacznie niewystarczający
- Całkowicie niewystarczający lub bezsenność trwa całą noc

67. Jak ocenia Pan/Pani jakość snu, niezależnie od czasu jego trwania?"

- Satysfakcjonująca
- Nieznacznie niesatysfakcjonująca
- Wyraźnie niesatysfakcjonująca
- Całkowicie niesatysfakcjonująca

68. Jakie jest Pańskie samopoczucie następnego dnia? *

- Dobre
- Nieznacznie gorsze
- Wyraźnie gorsze
- Wybitnie gorsze

69. Jaka jest Pańska sprawność psychiczna i fizyczna następnego dnia? *

- Niezaburzona
- Nieznacznie zaburzona
- Wyraźnie zaburzona
- Wybitnie zaburzona

70. Czy odczuwa Pan/Pani senność w ciągu dnia? *

- Nie
- Nieznaczną
- Wyraźną
- Bardzo nasiloną

Skala senności Epworth

Proszę określić jak dużo było w ostatnim czasie prawdopodobieństwo zaśnięcia w wymienionych sytuacjach. Jeśli Pan/Pani nie wykonywał/a tych czynności w ostatnim czasie, proszę je sobie wyobrazić i określić jak wpłynęłyby one na Pana/Panią

1. Siedzenie i czytanie
   - Duże
   - Średnie
   - Małe
   - Zerowe

2. Oglądanie telewizji
   - Duże
   - Średnie
   - Małe
   - Zerowe

3. Bierne siedzenie w miejscu publicznym (np. W teatrze, na zebraniu)
   - Duże
   - Średnie
   - Małe
   - Zerowe

4. Jako pasażer w samochodzie, jadąc przez godzinę bez odpoczynku
   - Duże
   - Średnie
   - Małe
   - Zerowe

5. Leżenie i odpoczywanie popołudniu
   - Duże
   - Średnie
   - Małe
   - Zerowe

6. W czasie rozmowy, siedząc
   - Duże
   - Średnie
   - Małe
   - Zerowe

7. Spokojne siedzenie po obiedzie bez alkoholu
   - Duże
   - Średnie
   - Małe
   - Zerowe

8. W samochodzie, podczas kilkuminutowego postoju (w korku, na czerwonym świetle)
   - Duże

- Średnie
- Małe
- Zerowe
