# Supplementary material for: Sleeping Disorders in Healthy Individuals with Different Dietary Patterns and BMI, Questionnaire Assessment
Source: Int J Environ Res Public Health. 2021 Nov 23;18(23):12285. doi: 10.3390/ijerph182312285 (PMC8656560; doi:10.3390/ijerph182312285)
Supplement: Supplementary file 1 [file ijerph-18-12285-s001.zip › File S2.pdf]

Content of the questionnaire

1. Diet (vegetarian, vegan, non-vegetarian)
2. How long have you been vegetarian? (Less than a month, 1-6 months, 6-12 months, 1-4 years, 5 years or more)
3. Gender (male, female)
4. Age (in years)
5. Weight (in kg)
6. Height (in cm)

7. Do you go to sleep everyday during working days at similar time? (Yes, No)
8. Do you get up everyday during working days at similar time? (Yes, No)
9. Do you go to sleep during non-working days at similar time as during working days? (Yes, No)
10. Do you get up during non-working days at similar time as during working days? (Yes, No)
11. How often do you perform naps? (Never, 1-3 times a month, once a week, few times a week, everyday, few times per day)
12. If you perform naps, how long they usually last? (In minutes)
13. Is your sleeping room completely dark? (Yes, No)
14.

KOMPAN QUESTIONNAIRE

1. How many meals do you usually eat? (1,2,3,4,5 and more)

Please recall your usual eating day during the last week and aswer the questions

2. What day was it? (Monday, Tuesday, Wednesday, Thursday, Friday, Saturday, Sunday)
3. How many meals did you eat that day? (Write a number)
4. How many times did you eat fruit or vegetables that day? (Write a number)
5. Did you eat fastfood that day? (Yes, No)
6. How many times did you eat fast food that day? (Write a number)

How often do you consume these products. During answering these questions take into consideration last year.
(Never, 1-3 times a week, once a week, few times a week, once a day, few times per day)

7. White bread
8. Wholewheat bread
9. White rice, white pasta, groats such as couscous, farina
10. Buckwheat, oats, wholewheat pasta, coarse-grained groats
11. Fast food e.g. fries, hamburgers, pizza, hot dogs
12. Fried food e.g. meat or flour meals
13. Butter
14. Lard
15. Milk including flavoured milk, cacao, coffee with milk
16. Fermented milk drinks e.g. youghurts, kefir
17. White chees including fromage
18. Cheese
19. Lunch meat, sausages, frankfuters
20. Red meat
21. White meat
22. Fish
23. Legumes
24. Fresh fruit

25. Vegetables
26. Confectioneries e.g. candies, cookies, cakes, chocolate bars, musli bars
27. Canned meat
28. Sweetened sparkling or still drinks e.g. Coca-Cola, Pepsi, Sprite, Fanta
29. Alcoholic beverages
